# Fast and Standardized Skin Grafting of Leg Wounds With a New Technique: Report of 2 Cases and Review of Previous Methods

**Published:** 2016-03-10

**Authors:** Nils Hamnerius, Ewa Wallin, Åke Svensson, Pernilla Stenström, Tor Svensjö

**Affiliations:** ^a^Department of Dermatology Malmö University Hospital, Malmö, Sweden; ^b^Department of Clinical Sciences Lund, Lund University, Sweden; ^c^Department of Plastic and Reconstructive Surgery, Malmö University Hospital, Malmö, Sweden; ^d^Department of Surgery, Central Hospital, Kristianstad, Sweden

**Keywords:** micrograft, skin transplantation, wound healing, leg ulcers, split thickness skin graft

## Abstract

**Background:** Chronic leg ulcers remain a challenge to the treating physician. Such wounds often need skin grafts to heal. This necessitates a readily available, fast, simple, and standardized procedure for grafting. **Objectives:** The aim of this work was to test a novel method developed for outpatient transplant procedures. **Methods:** The procedure employs a handheld disposable dermatome and a roller mincer that cut the skin into standardized micrografts that can be spread out onto a suitable graft bed. Wounds were followed until healed and photographed. **Results:** The device was successfully used to treat and close a traumatic lower limb wound and a persistent chronic venous leg ulcer. The donor site itself healed by secondary intent with minimal cosmetic impairment. **Conclusion:** The method was successfully used to graft 2 lower extremity wounds.

Both acute and chronic wounds are major clinical problems. On the basis of population studies conducted in Sweden, the point prevalence of leg or foot ulcers, of any cause, was reported to be 0.6%.^1^ The lifetime period prevalence in an adult population, that is, the number of adults who have ever had a leg or foot ulcer, was estimated to be 2.4%. This would be in Sweden, with its 9 million inhabitants, translate into approximately 50,000 people having an open leg ulcer and about 150,000 people who have a history of leg ulcers (open or healed).^1^ In chronic wounds, delayed or absent healing represents a major challenge. They are often complications of chronic illness such as diabetes, connective tissue disease, vascular insufficiency, or neuropathology. The resulting leg ulcers and pressure sores not only create a personal problem and a social inconvenience but sometimes also pose a threat to the limb and life of the patient.^2^^,^^3^ In some rare instances, the nonhealing wound develops neoplasia that may transform into a malignancy.^4^ For these reasons, restoration of an intact skin barrier is of critical importance. Treatment is often multifaceted and multidisciplinary. It involves correction, if possible, of the underlying disease, adequate wound bandaging, and, many times, supply of new skin to the wound, most commonly performed by autografting.

The history of skin grafting dates back to more than 2000 years ago, as it is believed to have been performed by the natives of India.^5^^,^^6^ More recently, Reverdin provided the first detailed description of pinch grafting in 1869.^5^^,^^6^ Later, in 1895, von Mangoldt^7^ described a method of creating small skin grafts by pulling a razor-like scraper over the skin to yield small skin particles. The resulting smear was applied to the surface of granulating wounds with reported success.^7^ The method was adopted by some clinicians,^5^ but it has never gained widespread use comparable with that of pinch grafting. Variants of the pinch grafts may also be obtained by utilizing a punch biopsy instrument (ie, a punch graft)^8^ or a specialized instrument, the trigger-fired pinch graft harvester.^9^ The method has been found to be particularly useful and efficient as a complement to conservative therapy of leg ulcers.^10^^,^^11^ Drawbacks, however, include poor cosmetic outcome, particularly at the donor sites, and a rather lengthy procedure.^12^ In 1958, Meek^13^ and, in 1959, Nyström^14^ presented an apparatus for mincing split-thickness skin grafts (STSGs) on a cutting apparatus that had several stainless steel lamellae run in parallel at a 1-mm distance from one another. The STSG was laid onto the lamella and cut into strips with a scalpel blade. The strips were then lifted, turned 90°, and repositioned over the lamella again, allowing it to be cut into small grafts with a size of 1 x 1 mm. The Nyström method was slightly meticulous, and a faster and more practical skin meshing technique was introduced by Vandeput and colleagues^15^ in 1963. The later method is the most widespread today and represents a cornerstone in the engraftment of large skin wounds with speed and predictable outcome. The method usually relies on nondisposable dermatomes and skin meshers, and for practical reasons their use are limited to services with equipped operating rooms. Wound practitioners encounter a large number of chronic wounds that have the potential to heal faster with a skin graft. In reality, many of these wounds are managed conservatively because of lack of surgical facilities and long referral times. We believe that more wounds would be grafted with skin if there were a fast and standardized procedure for autologous transplantation that would be manageable in an outpatient setting. Recently, a new transplant method that employs disposable instruments has been developed with this purpose. A case of a patient with burn injury treated with this technique was reported by Dr Danks and Lairet^28^ in 2010. In this article, we present 2 patients with leg wounds who were treated utilizing this new method.

## PATIENTS

Two patients, an 85-year-old woman with an acute, traumatic wound on the lower leg and a 54-year-old woman with a recurrent venous leg ulcer, were treated. Informed consent was obtained from both patients. The venous ulcer was included under a human study protocol approved by the regional ethical review board in Lund (www.epn.se). The patient with the acute wound arrived at the emergency department during a weekend and requested an immediate transplant procedure that could only be offered with the aid of the Xpansion MicroAutografting Kit, “Xpansion.”

## MATERIALS AND METHODS

**Figure 1:**
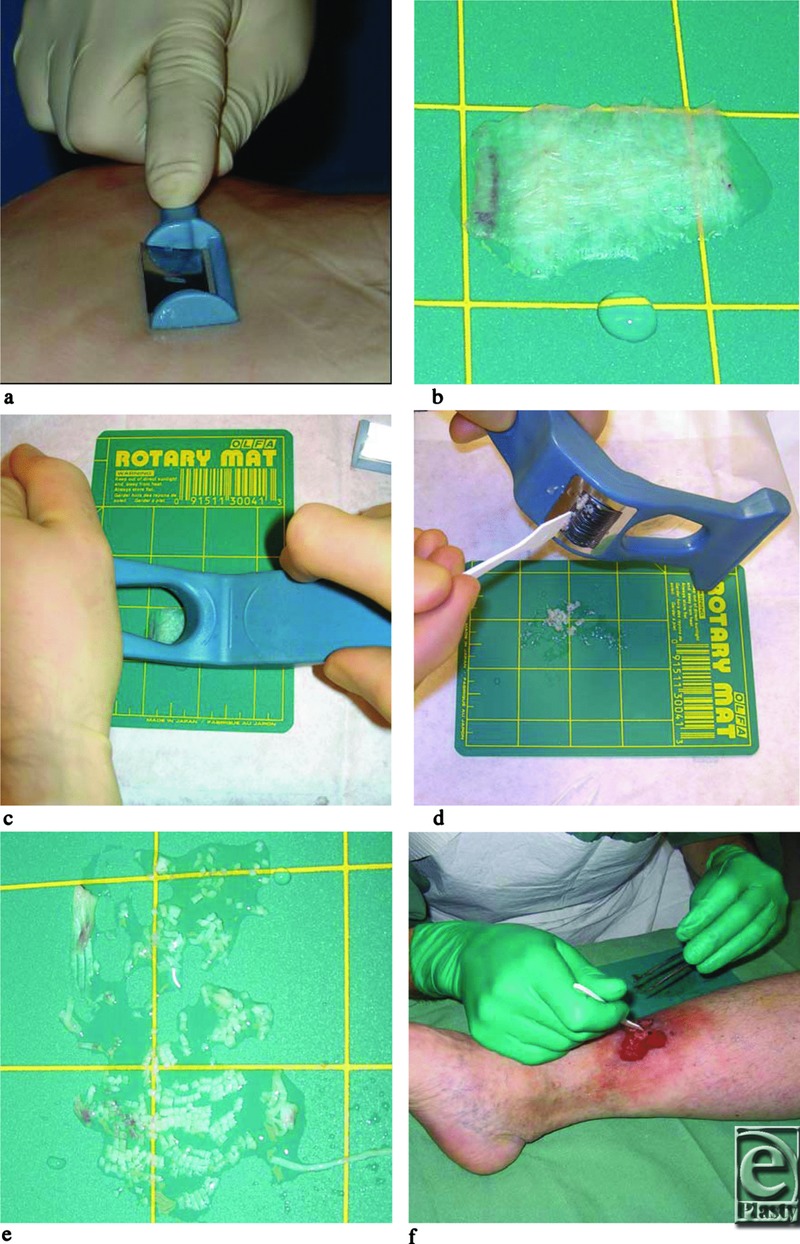
Transplantation procedure. (a) The handheld dermatome is run back and forth with mild pressure over the skin that has been moisturized with saline. (b) An approximately 4-cm^2^ STSG graft has been harvested and laid onto the cutting mat, which was wetted with a few droplets of saline. (c) The handheld mincer is run first once over the STSG and then once more in a 90° angle in relation to the first time. (d) The mincer has an array of circular blades mounted in parallel on an axle. Any skin fragments that stick to the mincer are removed with a spatula and transferred to the other fragments on the cutting mat. (e) Close-up of the skin fragments approximately 800 × 800 µm in size. Sometimes an extra run with the mincer is necessary to obtain complete mincing. (f) The skin fragments are kept together and spread out onto the wound bed. STSG indicates split-thickness skin graft.

The Xpansion MicroAutografting Kit (Applied Tissue Technologies LLC, Newton, Mass)* is a sterile, single-use disposable kit that contains a nonpowered handheld dermatome (Fig 1a) for harvesting a skin graft, a nonpowered handheld mincer for processing the graft into fine particles, as well as related supplies. The dermatome has a plastic handle with a surgical blade that is set at a fixed angle and exposure that allows for the dermatome to be held flat to the skin to harvest a graft (Fig 1b) approximately 0.32 mm (0.012 in) thick. The graft was transferred with forceps to a small cutting mat. The mincer has a plastic handle with an array of circular blades mounted in parallel on an axle. The device was rolled over the graft once to cut the graft into fine strips (Figs 1c and 1d), and again at a 90° angle to cut the strips into particles approximately 820 μm^2^ (Fig 1e). These particles were spread evenly across the wound bed (Fig 1f). No attempt was made to orient the particles in any particular direction since previous pig experiments performed by Hackl et al^29^ have shown that orientation of the skin grafts is not necessary. The grafts were covered with a silicone dressing (Mepitel; Mölnlycke Health Care, Gothenburg, Sweden) and a foam dressing (Allevyn, Smith and Nephew, Mölndal, Sweden, or Mepilex, Mölnlycke Health Care). The venous ulcer was also bandaged with a multilayer compression bandage system (Profore; Smith and Nephew). The acute wound was also covered with a silver dressing (Acticoat; Smith and Nephew) to prevent infection and a light compression bandage. Dressings were changed when necessary. Sterility was maintained during the initial procedure. Donor sites were located on the anterior thigh and were covered with a combined silicone foam dressing (Mepilex Border, Mölnlycke Health Care). Local anesthesia was managed with subcutaneous injection of Mepivakain (10 mg/mL) with epinephrine (5 µg/mL). After transplantation, the patients were asked to maintain a mild physical activity and to have their grafted legs elevated whenever sitting or lying.

## RESULTS

### An acute wound of the lower limb

**Figure 2:**
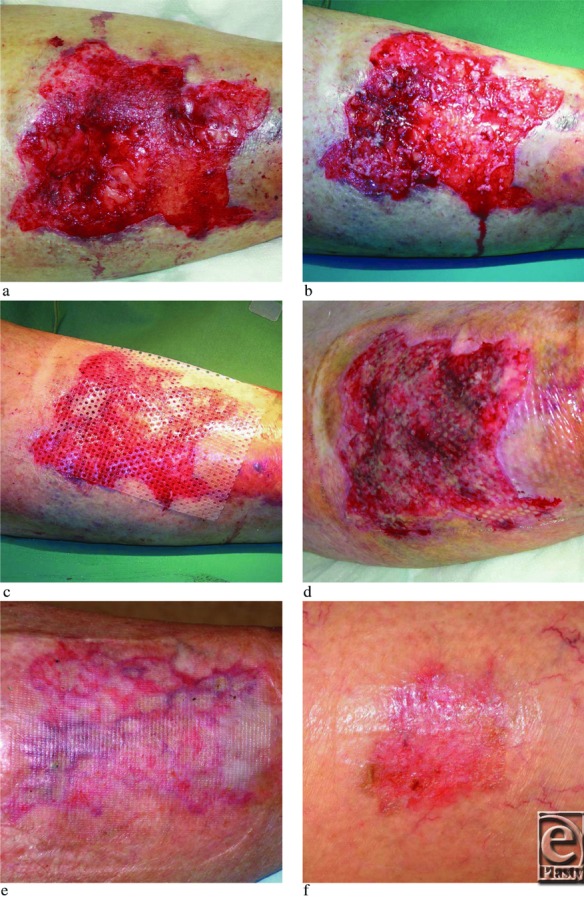
An acute wound measuring approximately 6 × 8 cm (width × length) with exposed muscle fascia, subcutaneous fat, and dermal tissue (a). (b) After suturing and transplantation. (c) Silicone dressing in place on the top of grafts. (d) Eight days posttransplantation with evidence of graft take seen as lighter colored epithelial islands. (e) Follow-up 6 weeks postgrafting showing a spotty pattern of the skin grafts separated by red scar tissue without any hypotrophy. (f) The donor site (approximately 2 × 2 cm) at 6 weeks also showing typical redness but no hypertrophic scarring. This redness usually disappears within a year.

A 85-year-old woman presented at the emergency department after having a fall in her apartment and during the fall she hit a furniture with a resulting wound measuring 6 × 8 cm (width × height) on her right anterior-lateral lower leg displaying exposed muscle, subcutaneous fat, and dermal tissue (Fig 2a). The wound was cleansed, subcutaneous tissues were adapted with sutures to cover the muscle, and the wound was grafted with skin harvested and processed with Xpansion (Figs 2b and 2c). The expansion rate was 1:12 (donor site 2 × 2 cm), and the procedure lasted approximately 15 minutes including local anesthesia and bandaging. The patient was admitted to the hospital for 12 days to allow for daily inspections, leg elevation, and the planning for home care. Eight days posttransplantation, graft take was evident (Fig 2c). The wound healed completely at day 25. A follow-up at 6 weeks showed a wound that remained healed and that exhibited a spotty pattern of incorporated micrografts (Fig 2d). The donor site also healed displaying redness but no hypertrophic scarring, typically what is seen in split-thickness skin donor sites at this time point (Fig 2e).

### A chronic venous leg ulcer

**Figure 3:**
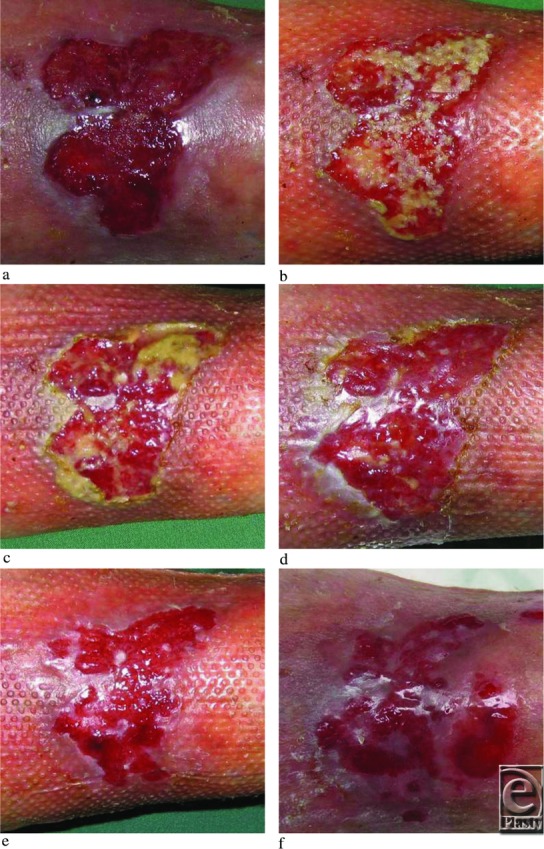

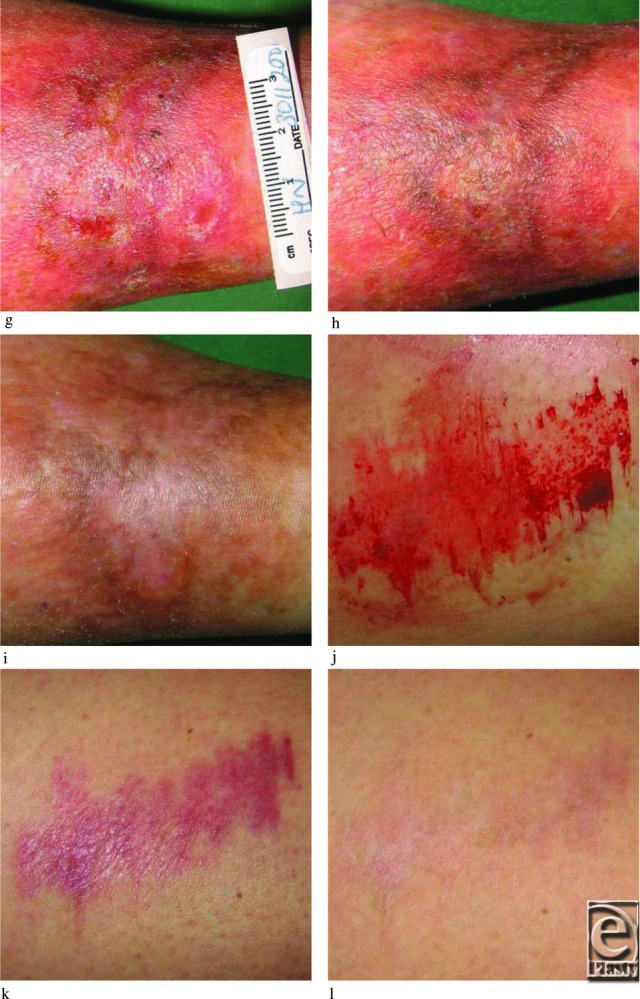
A venous ulcer transplanted with skin grafts. (a) The ulcer displaying complete granulation. (b) Three days postgrafting displaying grafts in place. (c) Ten days postgrafting and complicated by a mild infection (*Staphylococcus aureus*) with some pus. On day 17 (d) and day 24 (e), the wound appeared to be healing and small epithelial islands were sparsely distributed over the wound surface. (f) On day 31, healing has progressed significantly, and on day 38 (g), the wound was almost healed. (h) A completely healed wound on day 45. (i) Follow-up visit at 5 months and still healed. The redness has become less pronounced. (j) Donor site (approximately 2 × 6 cm) immediately after harvest, (k) at 24 days, and (l) at 5 months barely visible.

An otherwise healthy 54-year-old woman was referred to the Dermatology department with a pretibial ulcer on the right leg since 18 months (Fig 3a). Despite conservative therapy with compression bandages for 3 months, the ulcer failed to heal. The ankle brachial index was 0.8, blood pressure was 160/90 mm Hg, and the body mass index was 28.7. A marked insufficiency of the vena saphena magna was confirmed by Duplex investigation. There were no signs of deep venous insufficiency. Routine laboratory finding was normal (hemoglobin, erythrocyte sedimentation rate, albumin, blood glucose). The wound was clinically clean and exhibited granulation tissue; a routine wound swab showed only sparse growth of *Staphylococcus aureus*. Transplantation was performed with an expansion rate of 1:1.25 (donor site 2 × 6 cm, wound 3 × 5 cm) and the procedure lasted approximately 20 minutes including local anesthesia and bandaging. Routine visit 3 days postoperatively showed grafts in place (Fig 3b). Ten days postoperatively, there were signs of partial graft take (Fig 3c), but the wound also exhibited some pus and a scattered signs of folliculitis was seen in the surrounding skin. Treatment was initiated with isoxazolyl penicillin 500 mg 3 times daily for 10 days. On days 17 (Fig 3d) and 24 (Fig 3e), the wound showed healing and free of infection. On day 31, the surrounding skin once again presented with folliculitis as well as eczema. The wound, however, continued to heal as demonstrated by expanding epithelial islands and diminishing open wound area (Fig 3f). Treatment with isoxazolyl penicillin (in accordance with resistance pattern of cultured *S aureus*) and topical corticosteroids was started. On day 38, the wound almost healed (Fig 3g), and 45 days postoperatively, the wound healed completely (Fig 3h). At follow-up (5 months postoperatively), the wound had remained healed (Fig 3i), compression stockings class 2 were continuously used, and the donor site was hardly visible (Fig 3l) as compared with day 0 (Fig 3j) and day 24 (Fig 3k). The patient underwent foam sclerosing treatment of the insufficient vena saphena magna 7 months postoperatively. At 19 months, the patient continues with compressing stocking and her wound remains healed.

## DISCUSSION

This study presents a standardized and fast procedure for preparation of small skin grafts. The grafts, when laid onto an acute wound and a chronic wound, were associated with complete healing of the wounds. Similar and identical grafts have, in animal studies, been shown to reepithelialize full-thickness skin wounds.^16^ There are several mechanisms by which transplantation of small skin fragments might exert a positive effect on wound healing. First, graft take by incorporation and revascularization of the transplanted tissues may occur. This is very likely to have happened in our study, based upon the observations of isolated epithelial islands in the grafted wounds. Second, the supply of new cells, such as keratinocytes and fibroblasts, to the wound may, together with the proliferative stimulus of the wound environment, lead to proliferation, differentiation, and migration of the grafted skin. This mechanism is supported by the observation of epithelial islands in the grafted wounds that expanded and coalesced during the healing process in this study. It is also supported by reports that have shown accelerated healing in wounds transplanted with autologous cultured fibroblasts^17^ and keratinocytes^18^^-^20 as verified by sequential histologies and identification of the transplanted cells by gene marking. Such studies have concluded that the cells survive and incorporate into the tissues of the healing wound. Third, it is plausible that the grafted skin secrete one or several growth factors into the wound milieu, which are capable of augmenting the healing process. This presumes that the growth factors work in an autocrine and/or paracrine fashion and require that the cells of the transplanted tissues, or native cells in the wound environment, are capable of responding to growth factors or other cytokines released by the skin grafts. Our study could not determine whether such a mechanism was of any significance for the observed healing response, but this view is supported by other studies that have demonstrated accelerated healing of human leg ulcers transplanted with allogenic keratinocytes and fibroblasts.^21^^,^^22^ Such allogeneic cells are unable to survive in the long term due to incompatibility with the host,^22^ although it was initially reported otherwise.^21^

The minced skin technique used in this study is likely to be faster than the commonly used pinch grafting procedure. A direct comparison was however not performed. The STSG harvested in this study is also thinner than a typical pinch graft that often contains a significant portion of dermis. In our experience, a thin STSG donor site heals with minimal cosmetic impairment when evaluated one half to a year postoperatively. Pinch graft donor sites at a similar time point are, on the contrary, often highly visible as depigmented spotty areas exhibiting typical scar tissue.

In comparison with cultured autologous keratinocytes, we see several advantages with the procedure studied herein. First, the tissue is available immediately whereas keratinocytes need typically 3 to 4 weeks of culture to obtain a sufficient amount of cells.^23^^,^^24^ Second, keratinocyte culturing is very labor-intensive and relies on specialized laboratory equipment and facilities and therefore it is profanely expensive.^25^ Finally, keratinocyte grafting is a 2-stage procedure involving harvest of the donor skin necessitating a small surgical procedure and, at a later stage, when the cultures are ready, a transplant procedure must be performed. The advantage with cultured keratinocytes is, however, the virtually unlimited amounts of cells that may be obtained by serial propagation and subculture of the keratinocytes in vitro. For the relatively small wound, this is probably not necessary and it is quite possible that donor site size may be kept low by spreading the minced skin grafts extensively, thereby obtaining a high expansion factor. This remains to be studied in detail.

Another possible advantage of minced skin grafts is that they also contain a dermal support. The importance of a dermal tissues for the enhancement of transplantation of cultured keratinocytes has previously been stressed,^17^^,^^24^^,^^26^^,^^27^ and, if absent, the take rate of keratinocytes is often poor.

In relation to traditional split-thickness skin grafting and meshing,^15^ the minced skin technique offers the possibility of greater expansion. Greater expansion will, however, also lead to longer healing times and lower efficiency. We therefore do not believe that the minced skin procedure presented here would exclude traditional grafting, but we may consider it as a first line of treatment in the outpatient clinic as it offers a standardized and easily available treatment option for therapy-resistant skin ulcers.

## Footnotes

* The Xpansion MicroAutografting Kit is distributed by SteadMed Medical LLC, Fort Worth, Tex.
